# Lead (Pb^2+^) sorptive removal using chitosan-modified biochar: batch and fixed-bed studies†

**DOI:** 10.1039/c8ra04600j

**Published:** 2018-07-17

**Authors:** Narada Bombuwala Dewage, Ruth E. Fowler, Charles U. Pittman, Dinesh Mohan, Todd Mlsna

**Affiliations:** Department of Chemistry, Mississippi State University Starkville Mississippi 39762 USA tmlsna@chemistry.msstate.edu; School of Environmental Sciences, Jawaharlal Nehru University New Delhi 110067 India

## Abstract

Chitosan-Modified fast pyrolysis BioChar (CMBC) was used to remove Pb^2+^ from water. CMBC was made by mixing pine wood biochar with a 2% aqueous acetic acid chitosan (85% deacylated chitin) solution followed by treatment with NaOH. The characterizations of both CMBC and Non-Modified BioChar (NMBC) were done using diffuse reflectance infrared Fourier transform spectroscopy (DRIFTS), scanning electron microscopy (SEM), surface area measurements (*S*_BET_), elemental analysis, thermogravimetric analysis (TGA), differential scanning calorimetry (DSC) and ζ-potential measurements. Elemental analysis indicated that chitosan accounts for about 25% weight of the CMBC. The Langmuir maximum adsorption capacity of CMBC at pH 5 was 134 mg g^−1^*versus* 48.2 mg g^−1^ for NMBC at 318 K. CMBC column adsorption studies resulted in a capacity of 5.8 mg g^−1^ (Pb^2+^ conc. 150 mg L^−1^; pH 5; column dia 1.0 cm; column length 20 cm; bed height 5.0 cm; flow rate 2.5 mL min^−1^). CMBC removed more Pb^2+^ than NMBC suggesting that modification with chitosan generates amine groups on the biochar surface which enhance Pb^2+^ adsorption. The modes of Pb^2+^ adsorption on CMBC were studied by comparing DRIFTS and X-ray photoelectron spectroscopy spectra before and after Pb^2+^ adsorption.

## Abstract 2

Batch and fixed-bed column studies for the removal of lead (Pb^2+^) from aqueous solution by chitosan-modified pinewood biochar.

## Graphical abstract

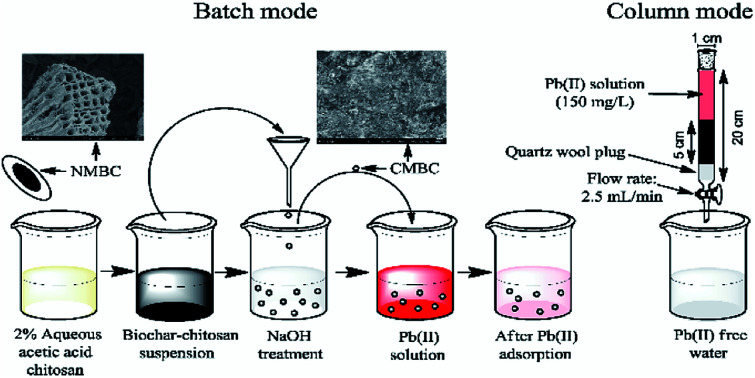

## 1. Introduction

Lead is a primary water pollutant observed in many developing countries.^1^ It is introduced to the environment in a variety of ways. The combustion of fossil fuels emits lead into the atmosphere and it is deposited back onto land, where it washes into nearby water systems. Acid mine drainage and discharge from industries that produce ceramics, glass, and acid batteries have been known to release lead into lakes and rivers.^2,3^ Lead can cause neurological, renal, hematological, endocrine, gastrointestinal, and cardiovascular problems in humans, and growth, cell division, water absorption and balance problems in flora and fauna.^4^ In humans, acute lead poisoning can cause severe kidney,^5^ brain, and neurological damage,^6^ while long term exposure can induce sterility and abortion.^7^ According to the World Health Organization (WHO), lead exposure accounts for approximately 143 000 deaths per year around the world. To reduce such health tragedies, the WHO and the American Water Works Association (AWWA) have determined the lead concentration in drinking water must fall below 10 μg L^−1^, while the U.S Environmental Protection Agency (EPA) set the maximum concentration in drinking water at 15 μg L^−1^.^2^

Many procedures have been developed to purify water contaminated with heavy metals. Aqueous solutions of these heavy metals have been treated by physical, chemical,^8^ and biochemical^9^ processes. However, adsorption techniques have become increasingly widely applied.^10^ Activated carbon, commonly used for adsorption, is expensive and selective for few contaminants.^11^ Thus, new, low cost adsorbents to remove heavy metals from aqueous solution are desirable.

Many low cost water purification methods have been studied.^12^ Viable choices include algae, which has a high lead uptake capacity (∼1.67 mmol g^−1^) and an almost unlimited ocean supply,^13,14^ red mud (a byproduct from aluminum industries composed mainly of iron oxide),^15^ and biochar.^16–18^ Fast biomass pyrolysis produces bio-oil and solid carbonaceous biochar, as a byproduct. Extensive research on biochar has proven its potential as a cheap and effective adsorbent to remove environmental contaminants from water.^2,19–23^ In an effort to improve biochar's adsorption performance, recent studies have focused on modifying the biochar surface.^24–30^

**Scheme 1:**
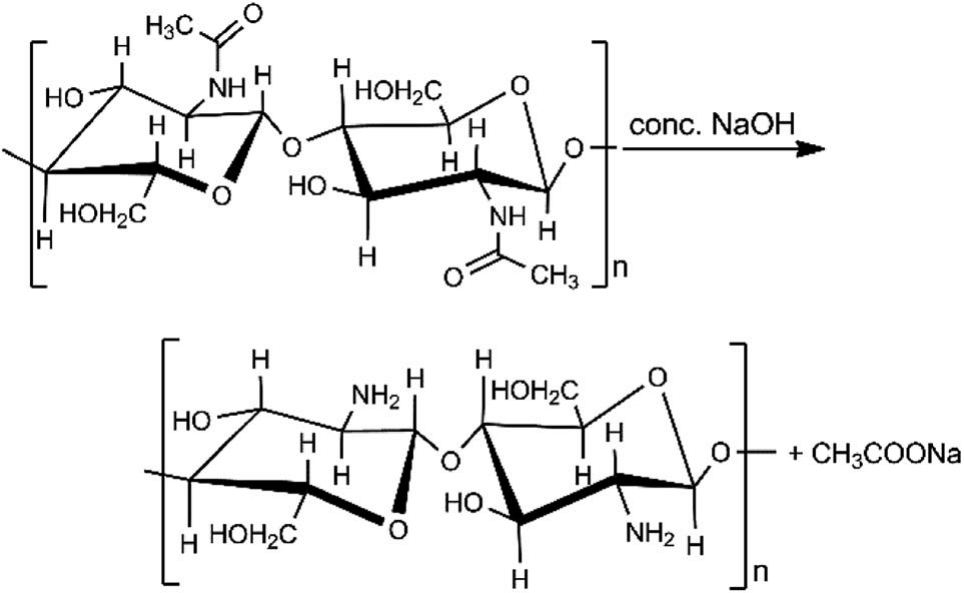
Chitin base hydrolysis to chitosan.

Chitosan is a low-cost, biodegradable, and non-toxic material that is created through the hydrolysis of chitin's amide functions to amine groups using alkali sodium hydroxide (Scheme 1).^31^ Chitin is found in shrimp and other crustacean shells. It is the most abundant renewable and biodegradable natural amino polysaccharide in the world, making chitosan potentially inexpensive. Chitosan is an excellent alternative adsorbent for heavy metal removal from aqueous solutions.^32–34^ Previously, lead adsorption on chitosan has been studied on chitosan hydrogel, chitosan/PVA hydrogel beads, and chitosan-coated sand.^35–37^ Therefore, combining chitosan and biochar could produce a novel material with increased lead ion uptake capacity above that of the biochar.^38^

Chitosan-modified bamboo, potato peel, and rice straw biochar have previously been used to study the sorption of Pb^2+^.^34,39,40^ However, the feedstock biomass used in these studies are not widely available in large quantities and reported Pb^2+^ adsorption capacities are relatively low compared to the literature reported values for other Pb^2+^ adsorbents. Another impediment to commercialization is that most adsorption data is from batch studies while wastewater treatment plants typically use fixed-bed columns for metal adsorption. The required column parameters for scaling up fixed-bed columns can be obtained through column studies. Thus, additional investigations are needed to develop high capacity and cost-effective chitosan-modified biochar adsorbents.

The main objectives of this work were to develop a new chitosan-modified biochar composite from pinewood, evaluate its batch and column sorption capacity, and elucidate sorption mechanisms for Pb^2+^. Pine wood was used as the feedstock to produce biochar due to its worldwide availability, cost effectiveness (available as a waste product from pulping and bio-oil industry) and high adsorption capacities.^41^ Chitosan-modified pine wood biochar could lead to low cost adsorbents, because both materials are cheap and widely available.

## 2. Experimental

### 2.1. Chemicals and equipment

All chemicals used were either GR or AR grades. They were purchased from Sigma Aldrich (Saint Louis, MO) unless otherwise specified. An aqueous stock solution of 1000 mg L^−1^ Pb^2+^ was prepared by dissolving Pb(NO_3_)_2_ in deionized (DI) water. Analytical grade chitosan (viscosity of 200–500 cP at 20 °C) 0.5 wt% in 0.5% aqueous acetic acid was acquired from VWR (Radnor, PA). This chitosan was derived from chitin by deacylating 85% of the parent chitin's amide groups. Complete deacylation is not required.

### 2.2. Preparation of pine wood biochar

The biochar used for this study was obtained as a byproduct of fast pyrolysis bio-oil production.^42^ The pine wood chips were pyrolyzed in a continuous auger-fed reactor where, after preheating, it was passed through the pyrolysis zone at 425 °C for 20–30 s. The heating protocol was previously described.^43^ The biochar was collected and washed with DI water several times to remove salt impurities and ash. Biochar was then ground, sieved to a uniform particle size distribution between 0.1 to 0.6 mm, oven dried at 110 °C for 12 h to remove moisture, and stored in a closed container for further use. Resulting non-modified pine wood biochar is hereafter referred as NMBC.

### 2.3. Preparation of chitosan-modified biochar

Chitosan-modified biochar was prepared as described by Y. Zhou *et al.*^34^ Briefly, 3 g of chitosan was dissolved in 180 mL of aqueous acetic acid (2%) followed by the addition of 3 g of biochar. The mixture was stirred for 30 min at ambient temperature. The biochar–chitosan suspension in aqueous acetic acid was then added dropwise to a 900 mL NaOH (1.2%) solution over approximately 2 h, and the resulting suspension was held for an additional 12 h. The solid was filtered through Whatman no. 1 filter paper. The chitosan-modified biochar was then washed with DI water to remove excess NaOH and oven dried for 24 h at 70 °C. The final weight of the dried sample was 4 g indicating the biochar had complexed 1 g of chitosan, giving a ∼25% w/w ratio of chitosan to the biochar in the chitosan-modified biochar. The resulting chitosan-modified biochar samples are hereafter referred as CMBC.

### 2.4. Biochar characterization

Diffuse reflectance infrared Fourier transform spectroscopy (DRIFTS) analysis (Thermo Nicolet 6700 FT-IR spectrometer) of the samples were obtained after grinding and pressing into a 5% by weight adsorbent KBr pellet. A total of 64 scans were taken from 4000 cm^1^ to 600 cm^−1^ with a resolution of 4 cm^−1^. Scanning electron microscopy (SEM) analysis was performed using a JEOL JSM-6500F FE-SEM operated at 5 kV. The biochar was applied to a carbon stub attached to carbon tape and then sputtered-coated under argon with a 5 nm layer of platinum. The biochar samples were then attached to a sample holder for SEM analysis. Transmission electron microscopy (TEM) analysis was carried out using a JEOL model 2100 TEM operated at 80 kV. About 10 mg of the samples were mixed with ∼0.5 mL of 100% ethanol, sonicated for about 4 min, deposited on carbon film on a 300 mesh copper grid, followed by a 24 h drying. Surface areas and pore size distributions were determined using nitrogen physisorption (BET) at 77 K with a NOVA 2200e surface area and pore analyzer purchased from Quantachrome Instruments. Isotherms were analyzed with Quantachrome's NovaWin software version 10.01. Thermogravimetric analysis (TGA) analysis was performed under air at a heating rate of 10 °C min^−1^ from 25 to 1000 °C for both NMBC and CMBC using a TA Instrument's Q50 thermogravimetric analyzer. Differential scanning calorimetry (DSC) analysis was performed under air at a heating rate of 10 °C min^−1^ from 25 to 550 °C for Chitosan, NMBC, and CMBC using a TA Instrument's Q20 differential scanning calorimeter. ζ-Potential measurements were conducted using a ZetaPALS instrument (Brookhaven Instruments Corporation (BIC), Holtsville, NY). The elemental C and N composition of both NMBC and CMBC determined by dry combustion using an ECS 4010 elemental combustion system CHNS–O (ECS 4010, Costect Analytical Technologies Inc.). X-ray photoelectron spectroscopy (XPS) analysis was performed using a Thermo Scientific K-Alpha XPS system equipped with a monochromatic X-ray source at 1486.6 eV corresponding to the Al Kα line.

### 2.5. Batch sorption studies

The effects of pH, contact time, and Pb^2+^ concentration on uptake were carried out using the batch adsorption method.^44^ Both kinetic and adsorption isotherm studies for Pb^2+^ were carried out at pH 5 and temperatures at 298, 308, and 318 K. A known amount of CMBC was added to 25 mL solutions of adsorbate containing 150 to 230 mg L^−1^ of Pb^2+^ from the 1000 mg L^−1^ Pb^2+^ stock solution prepared by dissolving Pb(NO_3_)_2_ into DI water. This range was selected based on the natural levels of lead in soil (range between 50 and 400 mg L^−1^). Samples were then shaken using a mechanical shaker at 200 rpm for 24 h (3 replicates were done for each test). Supernatants were then filtered through Whatman no. 1 filter paper. (As a test to determine if Pb^2+^ was adsorbed or retained on the filter paper, an aqueous Pb(NO_3_)_2_ solution (150 mg L^−1^) was filtered through the filter paper and the Pb^2+^ concentration in the filtrate was measured. It was found that the Whatman no. 1 filter paper can hold about 3.3% total wt of Pb^2+^ in solution. This was easily washed out from the filter paper with additional DI water). The Pb^2+^ concentration remaining in the filtrate was measured with Atomic Absorption Spectrometry (AAS) and the amount of Pb^2+^ removed by adsorption was calculated by:
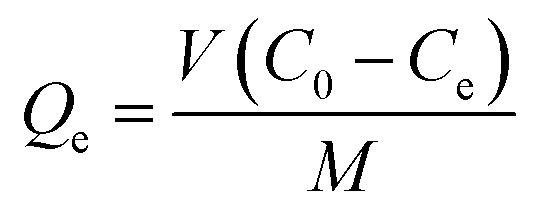
here *Q*_e_ is the amount of Pb^2+^ (mg) removed per gram of CMBC, *C*_o_ and *C*_e_ are the initial and equilibrium Pb^2+^ concentrations (mg L^−1^) in solution, *V* is the solution volume (L), and *M* is the CMBC weight (g).

### 2.6. Column sorption studies

The CMBC (1 g) was mixed with hot water producing a slurry that was packed into a glass column (20.0 × 1.0 cm) avoiding air entrapment. A small quartz wool plug was used to prevent any escape of CMBC. The bed height was 5.0 cm. The column was loaded with Pb^2+^ solution (150 mg L^−1^ and pH = 5.0) infiltrated downward through the column under gravity with a flow rate of 2.5 mL min^−1^. Effluents were collected and analyzed until Pb^2+^ concentration became close to the 150 mg L^−1^.

## 3. Results and discussion

### 3.1. Chitosan-modified biochar characterization

The FTIR spectra for chitosan, NMBC, and CMBC are shown in Fig. S1 (ESI†). The IR bands from 3300 to 3500 cm^−1^ are characteristic of N–H and O–H stretching vibrations. Chitosan (Fig. S1(a)†) shows the typical FTIR spectrum of chitosan with N–H and O–H vibrations centered in the 3300 to 3500 cm^−1^ regions and sp^3^-hybridized C–H stretching at 2871 cm^−1^. The bands at 1653 cm^1^ and 894 cm^−1^ are due to the N–H bending and N–H wagging respectively. The NMBC surface has a large number of alcohols and ethers (sp^3^-C–O and sp^2^-C–O stretching 1124–1205 cm^−1^), phenolic O–H (3200–3550 cm^−1^), and cyclic alkene (1566–1650 cm^−1^) which can be seen in the Fig. S1(b).† The CMBC surface contains amine and amide functional groups from chitosan and a few functional groups from the biochar including phenolic OH and carbonyls (Fig. S1(c)†).

**Fig. 1:**
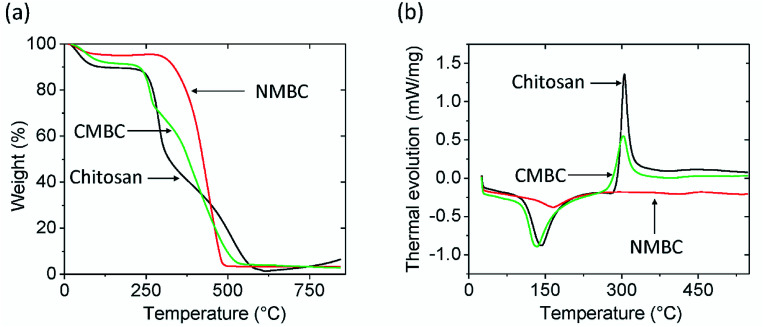
(a) Thermogravimetric analysis and (b) differential scanning calorimetry analysis for chitosan, CMBC, and NMBC at a heating rate of 10 °C min under air.

Thermogravimetric analysis (TGA) of chitosan, NMBC, and CMBC are shown in Fig. 1(a). All of these materials exhibit initial weight losses of 4–10% from 50–100 °C due to the loss of moisture. The NMBC starts to decompose just above 300 °C and loose about 90% of its mass by the time it reaches 480 °C 18 min later. CMBC exhibits two major weight drops. The first in the range of 220–290 °C due to chitosan decomposition. The second weight drop occurs from 300 to 530 °C and can be attributed to both biochar decomposition and the thermal reactions between the decomposing biochar and chitosan residues. The first weight drop accounts for about 20% of the weight of the sample, thus the CMBC TGA curve suggests that about 5% wt becomes chitosan residues reacted onto the decomposing char out of the initial chitosan ∼25% mass percentage present from the original synthesis of CMBC. Fig. 1(b) shows the DSC curves for the Chitosan and CMBC. The negative values of heat evolution observed beginning at 130 °C and continuing through 170 °C correspond to an endothermic phenomenon which could be due to the dehydration, nonbound water loss, and loss of low molecular weight organic compounds.^45,46^ Chitosan and CMBC show exothermic peaks around 300 °C which may relate to the decomposition of amine groups.^47^ A small endotherm for NMBC exists at ∼170 °C.

The elemental composition of biochar depends on a variety of factors such as feedstock type, pyrolysis residence time, temperature, and heating rate. The C and N composition of both NMBC and CMBC from combustion analysis are given in the Fig. S2.† The instrument response for N in CMBC indicates the presence of chitosan. No N was detected in the NMBC sample (Table S1†). The N in the CMBC accounts for about 4.6% of the weight of the CMBC indicating 25/75 chitosan/biochar wt/wt mixture. This compares well to the 25% chitosan composition derived from the weight gain obtained from the synthesis of CMBC. The ∼20% mass loss in the 220–290 °C portion of the TGA shows that about one fifth of the chitosan adsorbed still remained on the biochar at temperatures above 290 °C.

The N_2_ BET adsorption isotherm (Fig. S3†) and pore size distributions (Fig. S4†) of CMBC and NMBC demonstrate that more N_2_ is adsorbed by CMBC than by NMBC except in the low-pressure region. The single point surface area of CMBC (7.13 m^2^ g^−1^) is smaller than that of NMBC (10.5 m^2^ g^−1^), while the total average pore volume of CMBC (0.160 cm^3^ g^−1^) is higher than that of the NMBC (0.091 cm^3^ g^−1^). This is likely due to blockage of some small biochar pores by chitosan on CMBC, which leads to the high average total pore volume (Table S1†). Both biochars have a large amount of well distributed mesopores with different sizes (Fig. S4†) and negligible micropores (Table S1†).

The ζ-Potential of CMBC under different solution pH values is shown in Fig. S5.† ζ-Potential measures the surface charge present at different solution pH values. CMBC has point of zero ζ-Potential at pH 9.5. Below this pH, the CMBC surface charge is positive, while above pH 9.5 the surface is negative. At pH 7–12, Pb(OH)^+^ and Pb(OH)_2_ are the major lead(ii) species present in solution and both species will precipitate in this pH range.

The morphological structures of CMBC and NMBC were investigated before lead adsorption using SEM and TEM. SEM images (Fig. S6(a and b)†) of NMBC illustrate that the original mesoporous cell structure morphology of the pine wood largely remains after fast pyrolysis for 20 to 30 s at 425 °C. Areas of the surface pore structure appear to be blocked or partially obscured by deposited chitosan in the CMBC (Fig. S6(c and d)†).

TEM/EDX analysis reveals no nitrogen on NMBC (Fig. S7(a)†) *versus* substantial surface region nitrogen (5.4 wt%) on CMBC (Fig. S7(b)†) for the selected sample area, confirming chitosan modification in this surface region. Element mapping of N clearly shows that the N is uniformly and densely distributed over the region mapped on the CMBC surface.

### 3.2. Batch sorption studies

#### 3.2.1. Effect of pH on adsorption

**Fig. 2:**
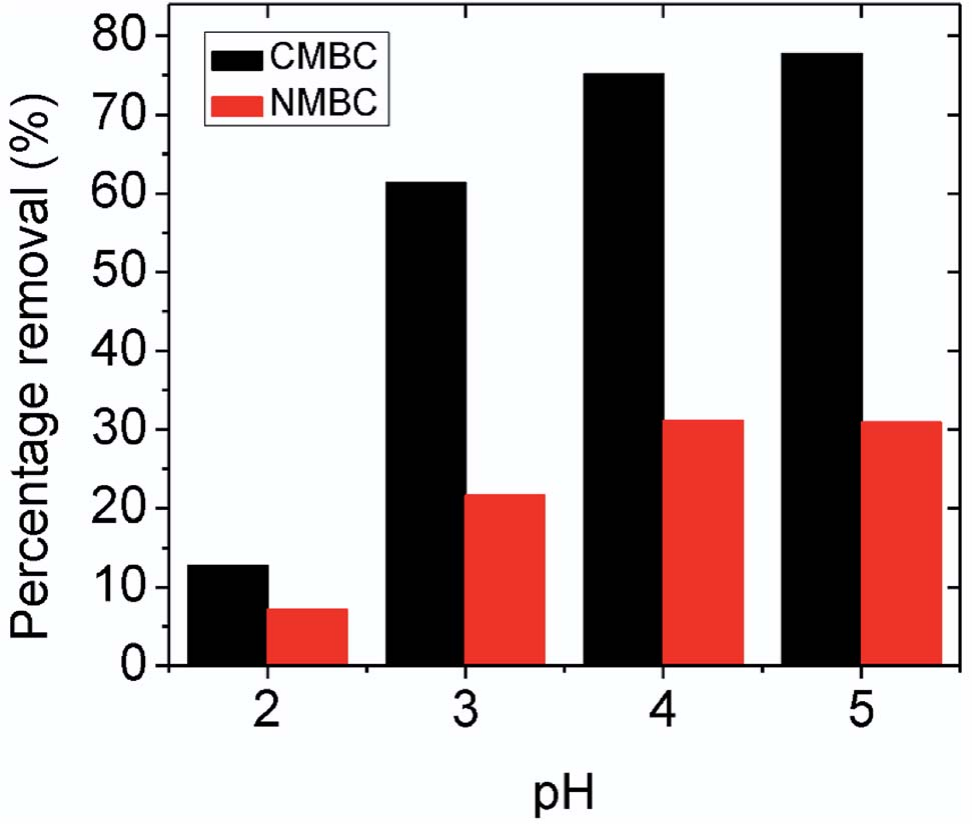
Percentage removal of lead at equilibrium by NMBC and CMBC at different pH values by 0.05 g adsorbent in 25 mL of aqueous Pb(NO_3_)_2_, concentration = 150 mg L^−1^ at 25 °C.

The Pb^2+^ adsorption of CMBC and NMBC at different initial pH values is shown in Fig. 2. The maximum pH studied was 5 to avoid Pb^2+^ precipitation (another remediation technique).^48^ Pb^2+^ removal by CMBC at equilibrium is more than two times higher than that of NMBC at every solution pH's except at pH 2. Lead removal by both CMBC and NMBC increases with increased pH, as expected from the ζ-potential data, which shows decreasing positive surface charge with increased pH. However, the maximum removal of Pb^2+^ is at pH 5, where the net surface charge is positive and lead ion repulsion still exists. Therefore, the mechanism of Pb^2+^ adsorption on CMBC must include specific non-electrostatic interactions to achieve this removal. Possible mechanisms include specific sorption by amine group coordination of Pb^2+^, physical attraction, precipitation, and reduction.

#### 3.2.2. Adsorption mechanisms

##### 3.2.2.1 pH dependent mechanism

The possible Pb^2+^ ion adsorption sites on CMBC include the chitosan amino groups, biochar carboxylic acid groups and the aliphatic hydroxyl groups on chitosan and phenolic biochar hydroxyls. Many studies have explored the effect on the chitosan amine group in metal chelation and reported that the C, O, and H atoms are not involved in the lead adsorption.^34,35^ In this study, the maximum percentage removal was observed at pH 5 (see Fig. 2), where the surface charge is positive (below the point of zero ζ-potential). The surface coating of chitosan on the CMBC surface in water undergoes a pH dependent protonation equilibrium at its primary amine functions.^49^ Since the chitosan used had 85% of its –NHCOCH_3_ functions hydrolyzed to amine groups, 85% of its monosaccharide rings contain primary amine functions.

**Scheme 2:**
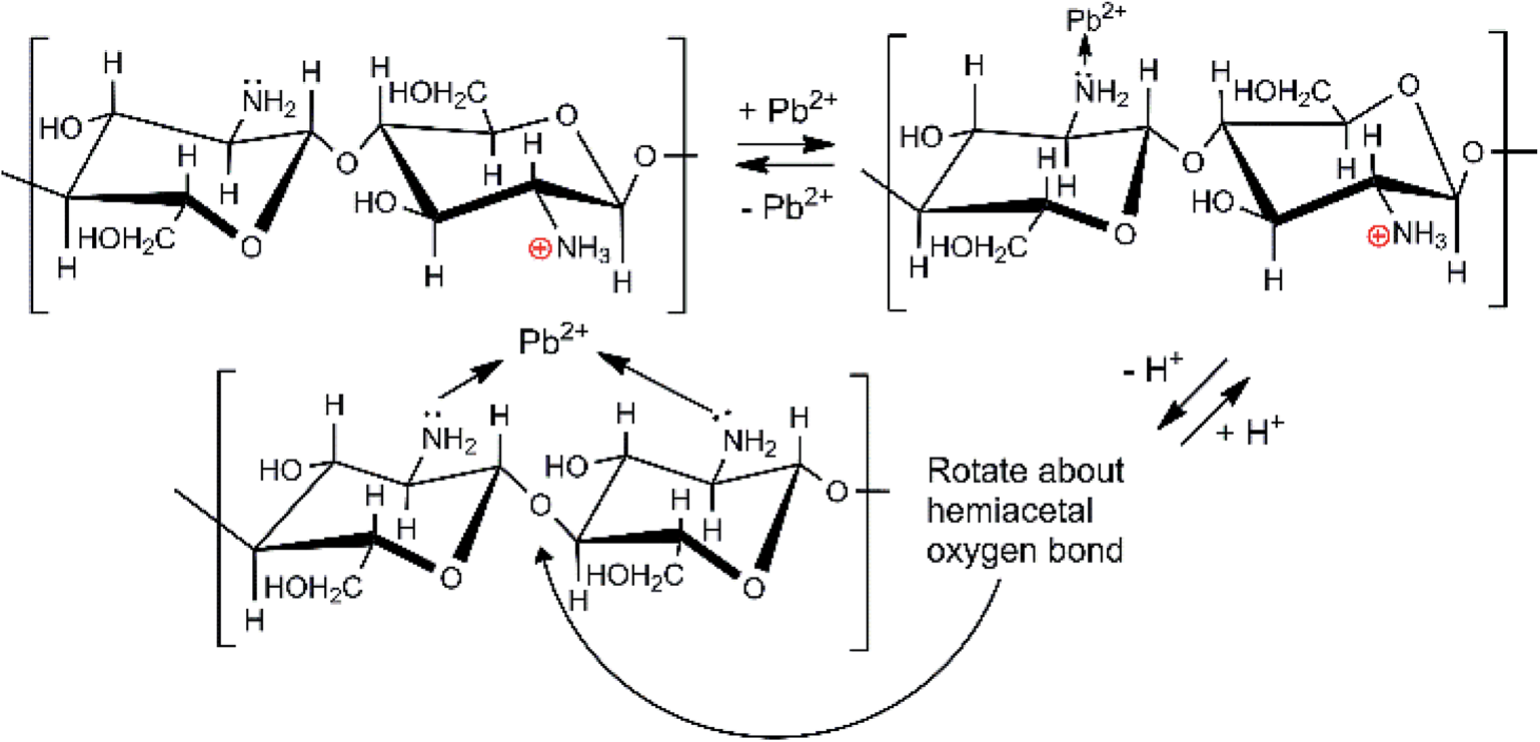
Chitosan's amino group coordination with Pb^2+^ ions.

Depending on the solution pH, chitosan's basic –NH_2_ groups will undergo protonation to –NH_3_^+^. As the pH rises, the fraction of amine sites that is protonated drops, as illustrated in Table S2.†^50^ Chitosan adsorbs Pb^2+^ ions by amino group coordination to Pb^2+^ ions. This is shown in Scheme 2. As the pH drops (more acidic), less Pb^2+^ is adsorbed. Strong acids should increasingly remove Pb^2+^ from the adsorbent.

As noted in Table S2,† at pH 5 only about 5–6% of the chitosan amine groups are not protonated. Nevertheless, every monomer ring has an –NH_2_ or –NH_2_^+^ function. Thus, even at pH 3, the chitosan can adsorb a substantial amount of Pb^2+^ by amine coordination.

Surface carboxylic acid sites on the biochar can also complex Pb^2+^ as they do with Ca^2+^ and Mg^2+^ ions *via* chelation, 2RCOO^−^ + Pb^2+^ → [(RCOO^−^)_2_Pb^2+^].^24^ These are acidic sites (p*K*_a_ ∼4.20–4.75) because their carboxylate conjugate bases are stable and complex metal cations like Pb^2+^.

##### 3.2.2.2 Mechanistic studies by FTIR and XPS

**Fig. 3:**
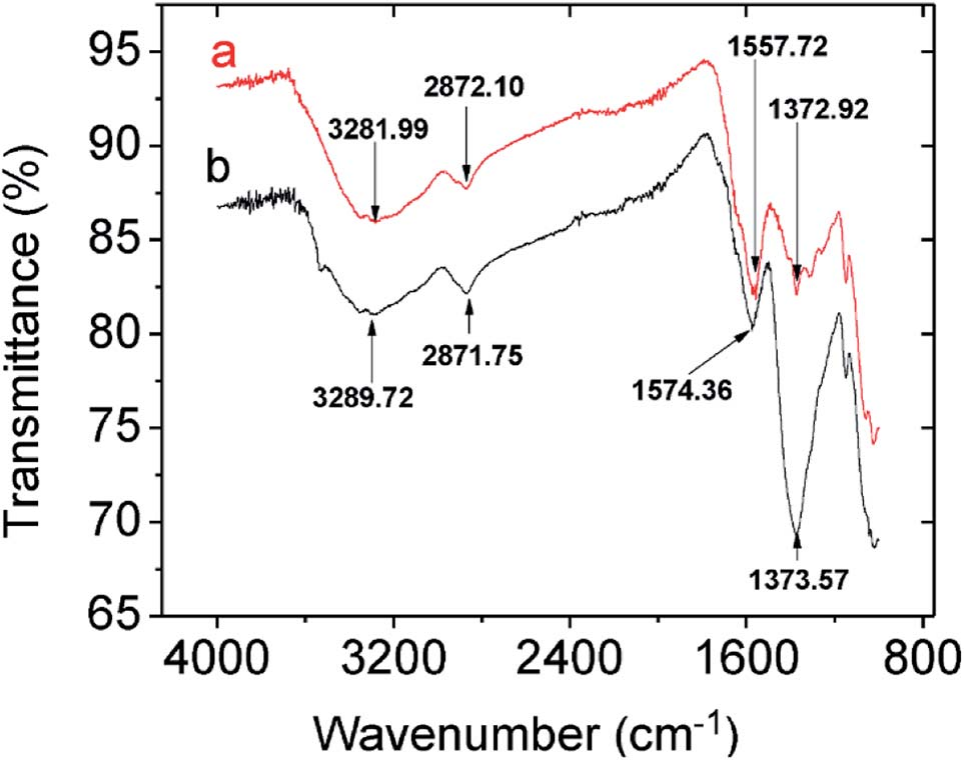
FTIR spectra of CMBC (a) before lead adsorption (b) after lead adsorption.

**Fig. 4:**
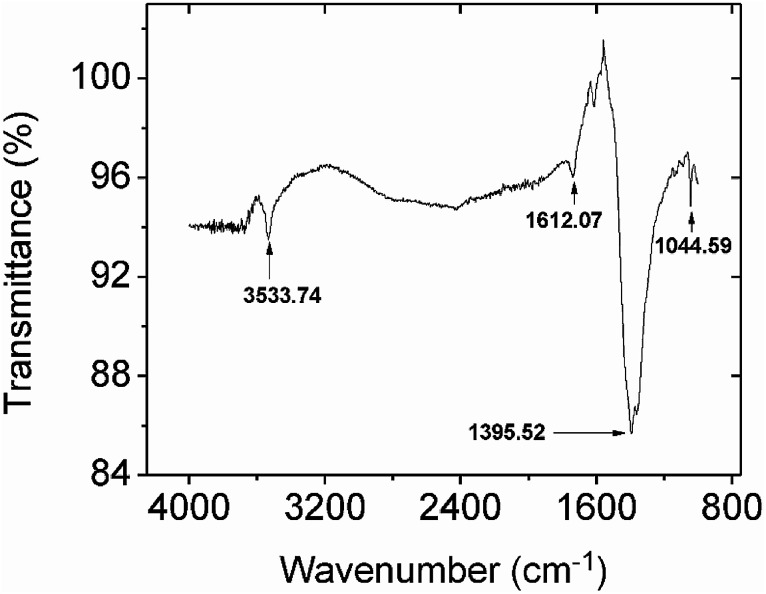
The subtracted FTIR spectrum from spectra obtained before and after lead adsorption onto CMBC.

Comparing the FTIR spectra before and after Pb^2+^ adsorption suggests the nature of Pb^2+^ adsorption occurring on the CMBC surface (Fig. 3). A small N–H vibration band shift from 3282 to 3290 cm^−1^ is observed after Pb^2+^ adsorption. This indicates the attachment of Pb^2+^ to the N group affecting the N–H vibration. This observation is in parallel to a previous study where binding of iron ions to NH_2_ group of chitosan shifted the N–H bending vibration from 1638 to 1681 cm^−1^.^51^ Moreover, the subtracted spectrum (Fig. 4) clearly indicates the transmittance drops in the 3534, 1612, 1396 and 1045 cm^−1^ regions, which are related to the N–H stretching, bending, scissoring and wagging, and C–N stretching bands respectively. All of these changes could be attributed to Pb^2+^ ions binding onto the amino groups. These results are in accord with those already reported for the Pb^2+^ adsorption on chitosan/PVA hydrogels.^35^

XPS is a powerful tool for studying the surface/near surface (∼100 Å) chemistry of a material. Fig. S8(a)† shows the chitosan XPS spectrum and Figs. S8(b) and (c)† show the XPS spectra for CMBC before and after the lead adsorption. Nearly identical peak positions with different intensities were observed with chitosan and CMBC illustrating the successful coating of chitosan on the biochar surface. Following adsorption, the 4f peak for lead appears in Fig. S8(c)† indicating the adsorption of lead on the surface of CMBC.

**Fig. 5:**
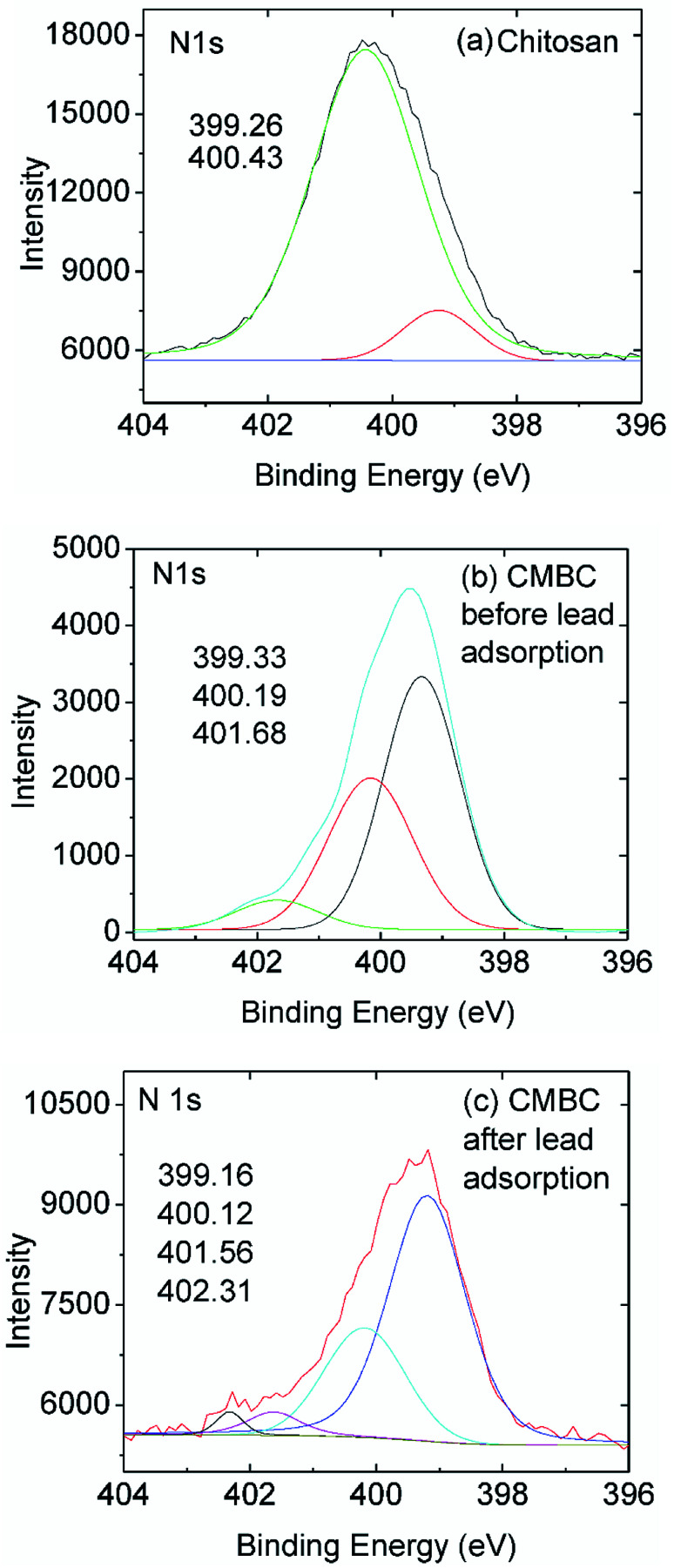
High resolution N 1s XPS spectra of (a) chitosan and (b) CMBC before and (c) CMBC after lead adsorption.

High resolution XPS spectra of chitosan and CMBC before and after lead adsorption (Fig. 5) revealed the presence of two N 1s peaks upon deconvolution. Both amide nitrogen at 400.43 eV and amine nitrogen at 399.26 eV are present for this ∼85% deacylated chitin. The peak at binding energy 400.43 eV is attributed to the N atom in the R–NHCOCH_3_ (amide) group, and the peak at binding energy 399.26 eV is attributed to the N atom in the R–NH_2_ group (chitosan).^52^ Each peak's relative intensity is proportional to the percentage of each component in that material (Fig. 5(a)).

The XPS N 1s spectrum of CMBC has three resolved peaks with binding energies of 399.33, 400.19, and 401.68 eV (Fig. 5(b)). The binding energy of the first two match the XPS spectrum of chitosan. This indicates that the functional groups of chitosan remained on the biochar surface even after the chitosan coating modification of the biochar. The additional peak at a binding energy of 401.68 is attributed to the N atom in the protonated R–NH_2_ group confirming that a positive charge is present on the biochar surface as shown from the ζ-potential. After lead adsorption, CMBC exhibits four N 1s peaks at binding energies of 399.16, 400.12, 401.56 and 402.31 eV (Fig. 5(C)). The first three peaks belong to amine, amide, and protonated amine N atoms, similar to the previous XPS spectrum of CMBC before lead adsorption. The fourth N peak at 402.31 eV is attributed to amine functions coordinated to Pb^2+^ sites [ R–NH_2_ → Pb^2+^ and/or (R–NH_2_)_2_Pb^2+^ chelated sites].

#### 3.2.3. Sorption dynamics

The effect of temperature on lead adsorption was studied using 2 g L^−1^ of CMBC, 150 mg L^−1^ Pb^2+^, shaking for 24 h, pH of 5 at 298, 308, and 318 K. Significant lead adsorption was observed within 1 h and equilibrium was reached after approximately 6 h (Fig. S9†). The amount of lead adsorption at 298 and 308 K were similar, while greater adsorption occurred at 318 K, suggesting endothermic behavior. All kinetic studies were carried out over 6 h to ensure that equilibrium was achieved.

The effect of Pb^2+^ concentrations on adsorption was studied using 25 mL of 150, 175, and 230 mg L^−1^ lead solutions, 2 g L^−1^ of CMBC, and 6 h shaking at pH 5. Adsorption capacity increased with increasing initial lead concentration, and a significant adsorption capacity increment was observed upon increased Pb^2+^ from 175 mg L^−1^ to 230 mg L^−1^ (Fig. S10†).

#### 3.2.4. Adsorption kinetics

The pseudo first order linear kinetics model^53^ was fit to
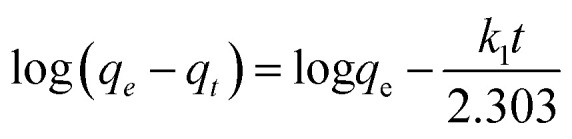
where, *q*_*t*_ is the amount of lead adsorbed at time “*t*”, *q*_e_ is the amount adsorbed at equilibrium, and *k*_1_ (h^−1^) is the first order adsorption rate constant. Plots of log(*q*_e_ − *q*_*t*_) *versus t* can be found in Fig. S11† for lead solutions of 150, 175, and 230 mg L^−1^ at 298, 308, and 318 K. The parameters, correlation coefficients (0.915–0.970) for the first order kinetics model and the calculated *versus* observed *q*_e_ values (Table S3†) were not satisfactory. Thus, pseudo second order fittings were conducted.

The linear version of the pseudo second order kinetics model^54^ is given by,
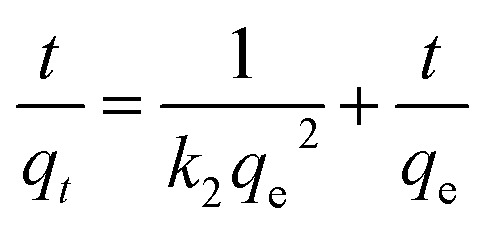
where, *q*_*t*_ is the amount of lead adsorbed at time “*t*”, *q*_e_ is the amount adsorbed at equilibrium, and *k*_2_ (h^−1^) is the second order adsorption rate constant. Linear plots of *t*/*q*_*t*_*vs. t* (slope of 1/*q*_e_) are shown in Fig. S12.† The second order kinetic model parameters for the lead solutions of 150, 175, and 230 mg L^−1^ at 298, 308, and 318 K are provided in Table 1. The correlation coefficients for the second order kinetics model are all larger than 0.991, and the calculated *q*_e_ values and the experimental *q*_e_ values matched well. We conclude lead adsorption on CMBC is second order.

**Table 1:** Pseudo-second order parameters for lead adsorption (pH = 5) at (a) 298 K, (b) 308 K, and (c) 318 K for Pb^2+^ concentrations of 150, 175, and 230 mg L^−1^, using 2 g CMBC/L

Pseudo-second order parameters on CMBC					
Temp. (K)	Initial conc. (mg L^−1^)	*q*_e_ exp. (mg g^−1^)	*q*_e_ calc. (mg g^−1^)	*k*_2_ (*g* m g^−1^ h)	*R* ^2^
298	150	63.8	62.5	0.085	0.999
	175	79.6	71.4	0.049	0.991
	230	88.3	83.3	0.072	0.996
308	150	63.6	66.7	0.056	0.998
	175	76.1	83.3	0.048	0.999
	230	91.3	90.9	0.061	0.996
318	150	73.4	76.9	0.084	0.999
	175	79.5	83.3	0.072	0.998
	230	96.1	100	0.100	0.999

#### 3.2.5. Adsorption isotherm models

The lead adsorption on CMBC was studied using different adsorption isotherm models. Adsorption isotherm data were collected at 298, 308, and 318 K, Pb^2+^ concentrations from 3–350 mg L^−1^, using a 12 h shaking period. The data was evaluated using the two parameter Langmuir^55^ and Freundlich,^56^ models applying a nonlinear regression calculated employing OriginPro 2016 software (Fig. S13†).

The endothermic behavior of lead adsorption on CMBC is further indicated by the adsorption isotherm studies, which show greater amounts of adsorption at higher temperatures. The isotherm parameters are shown in Table 2. Pb^2+^ uptake is endothermic for oak wood and oak bark biochar,^2^ as well as pine wood and rice husk biochar.^57^ Higher temperatures also favored significant increases of Cu(ii) and Zn(ii) adsorption onto corn straw and hardwood biochars.^58^ The chitosan coating renders CMBC surfaces amine-group rich, hence chemically different than biochar surfaces.

**Table 2:** Langmuir and Freundlich model parameters for Pb^2+^ adsorption on CMBC

Isotherm parameters		298 K	308 K	318 K
Langmuir	*Q* ^0^ (mg g^−1^)	50.5	103	134
	*b*	0.0791	0.0149	0.0113
	*R* ^2^	0.996	0.994	0.988
Freundlich	*K*_f_ (mg g^−1^)	13.5	6.58	6.33
	*n*	3.82	2.09	1.93
	*R* ^2^	0.999	0.996	0.994

The Langmuir model provided a better fit than the Freundlich model with *R*^2^ values all greater than 0.988. This favors a proposed monolayer lead adsorption mechanism (an assumption in the Langmuir model) for Pb^2+^ ion binding. This observation is consistent with previous studies of heavy metal ion adsorption onto amine-functionalized materials.^59^ In a previous study of Pb^2+^ adsorption on magnetic (containing Fe_3_O_4_) char, diffusion controlled adsorption was suggested at low Pb^2+^ concentrations and monomolecular adsorption at high Pb^2+^ concentrations.^60^

Lead adsorption on CMBC is mainly due to the chitosan amine functions coordinating to Pb^2+^ with additional adsorption by the biochar. The CMBC Langmuir adsorption capacity is 134 mg g^−1^ at 318 K compared to a value of 48.2 mg g^−1^ for the NMBC, despite the fact that CMBC has only 68% of NMBC's surface area. This value is also much higher than previously reported biochar capacities for the lead adsorption (Table 3).

**Table 3:** Comparison of CMBCs' Pb^2+^ adsorption capacity with those of pine wood, bamboo, potato peel biochar, chitosan, and activated carbon (carbon F-400)

Adsorbent	pH	Temp. (K)	Conc. range (mg L^−1^)	Surface area (m^2^ g^−1^)	Pb^2+^ adsorption capacity (mg g^−1^)	Pb^2+^ adsorption capacity (mg m^−2^)	Ref.
Chitosan-modified pine wood biochar (CMBC)	5.0	298	50–350	7.13	50.5	7.08	This study
		308			103	14.4	
		318			134	18.8	
Non-modified pine wood biochar (NMBC)	5.0	318	10–350	10.5	48.2	4.59	This study
Chitosan-modified bamboo biochar (BB-C)	5.5	298	2–100	166.9	14.3	0.085	34
Chitosan-modified potato peel biochar	Not available	Not available	Not available	Not available	0.147	—	40
Chitosan and pyromellitic- modified rice straw biochar (CPMB)	5	298	5–500	62.6	9.24	0.15	39
		303			11.91	0.19	
		308			13.93	0.22	
Chitosan	4.5	293	10–1000	Not available	0.558 (mmol g^−1^)	—	61
Carbon F-400	5.0	278	0.125–100	984	44.3	0.0450	2
		298			30.1	0.0306	
		323			25.2	0.0256	

### 3.3. Fixed-bed studies

**Fig. 6:**
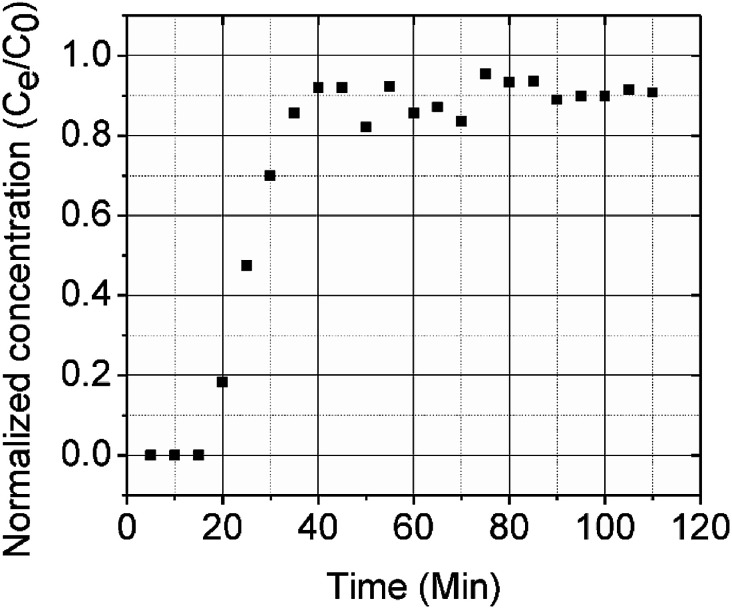
CMBC breakthrough curve (Pb^2+^ concentration = 150 mg L^−1^, pH = 5).

A column (length = 20 cm; diameter = 1 cm) packed with 1 g of CMBC was used to study Pb^2+^ sorption. A gravity propelled Pb^2+^ solution (150 mg L^−1^, pH = 5) ran through the column at flow rate of 2.5 mL min^−1^ until column saturation was reached. A breakthrough curve using the normalized concentration (*C*_e_/*C*_o_) *versus* time is shown in Fig. 6.

The column parameters were obtained by analyzing the column experimental data. Following mathematical expressions were used.^62^1
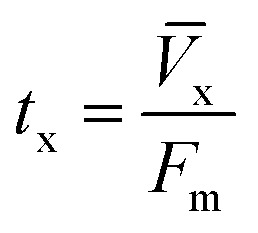
2
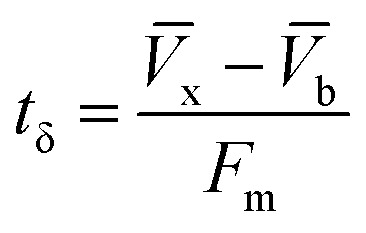
3
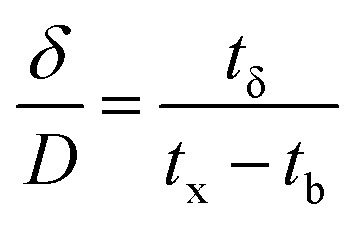
4
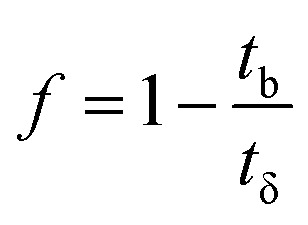
5
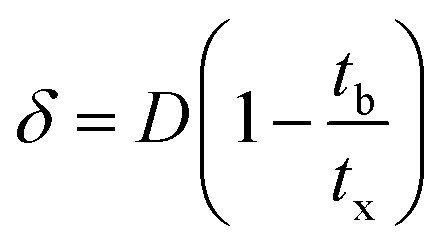
6
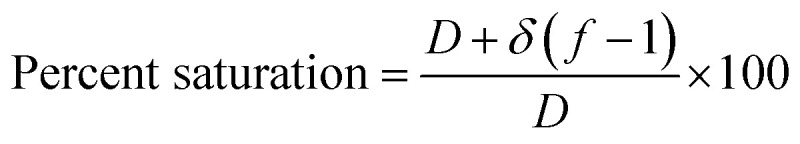
7
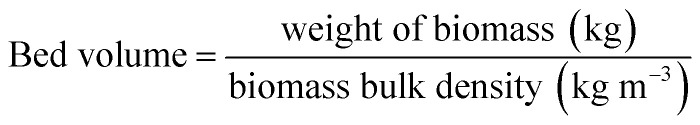
8
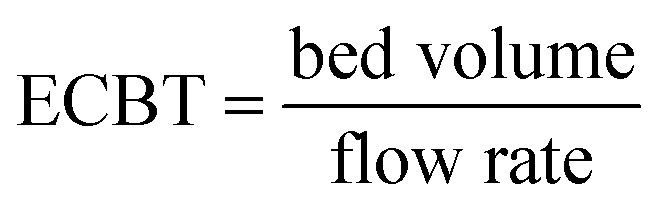
where *t*_x_ is the total time involved for primary adsorption zone establishment, *t*_δ_ is the time for the primary adsorption zone (PAZ) to move down to its length, *δ* is the length of the primary adsorption zone, *f* is the fractional capacity, *D* is the bed depth, *t*_b_ is the time required for initial PAZ formation, *F*_m_ is the mass rate of flow to the adsorber, *V*_b_ and *V*_x_ are the total effluent mass quantity per unit adsorbent area at the breakpoint, and the total effluent mass quantity per unit adsorbent area when adsorbent is approaching saturation, respectively. *C*_x_ is the effluent concentrations at *V*_x_. The percent saturation of column at breakthrough, bed volume, and the empty-bed-contact-time (EBCT) were also calculated. Table 4 summarizes the values of all the column parameters. The obtained column capacity (5.8 mg g^−1^) is significantly lower than batch adsorption capacity (134 mg g^−1^). This observation is similar to those reported for Pb^2+^ adsorption by tea waste and modified activated carbon.^7,63^

**Table 4:** Fixed bed parameters for Pb^2+^ adsorption by CMBC

Parameters	Values
Column diameter (cm)	1.0
Column radius (cm)	0.5
Bed volume (cm^3^)	3.9
Column capacity (mg g^−1^)	5.8
Breakpoint capacity (mg g^−1^)	0.16
*C*_o_ (mg mL^−1^)	0.15
*C*_x_ (mg mL^−1^)	0.13
*V*_b_ (mg cm^−2^)	6.5
*V*_x_ (mg cm^−2^)	12.1
*t*_x_ (min)	110
*F*_m_ (mg cm^−2^ min)	0.11
*D* (cm)	5.0
*t*_b_ (min)	55
*t*_δ_ (min)	50.5
*F*	−0.09
*δ* (cm)	2.5
EBCT (min)	1.6
Saturation (%)	45.50

## 4. Conclusions

Pine wood biochar was modified through a surface deposition of chitosan which greatly increased the Pb^2+^ adsorption capacity. Chitosan deposition allowed rapid flow through columns or beds due to the biochar particle sizes. Maximum lead removal occurred at pH 5 and 318 K displaying the pH dependent and endothermic behavior for lead adsorption. Pseudo-second order kinetics provided the best fit with regression coefficients of 0.991 or greater. Sorption was evaluated from 298 to 318 K using the Freundlich and Langmuir isotherm models and best fit observed with the Langmuir model. A fixed-bed column study for Pb^2+^ showed a column capacity of 5.8 mg g^−1^. The Pb^2+^ adsorption mechanism on CMBC biochar was mainly controlled by the coordination between the chitosan amine groups and Pb^2+^ ions based on FTIR and XPS evidence. CMBC has great potential for heavy metal contaminant removal from aqueous solution.

## Conflicts of interest

There are no conflicts to declare.

## Supplementary Material

RA-008-C8RA04600J-s001
