# Supplementary material for: Lead (Pb2+) sorptive removal using chitosan-modified biochar: batch and fixed-bed studies
Source: RSC Adv. 2018 Jul 17;8(45):25368–77. doi: 10.1039/c8ra04600j (PMC9082581; doi:10.1039/c8ra04600j)
Supplement: RA-008-C8RA04600J-s001 [file RA-008-C8RA04600J-s001.pdf]

Electronic Supplementary Material (ESI) for RSC Advances.
This journal is © The Royal Society of Chemistry 2018

Supplementary data

Lead (Pb^{2+}) sorptive removal using chitosan-modified biochar: batch and fixed-bed studies

Narada Bombuwala Dewage,[a] Ruth E. Fowler [a] Charles U. Pittman Jr.,[a] Dinesh Mohan[b] and Todd Mlsna*[a]

[a] Department of Chemistry, Mississippi State University, Starkville, Mississippi 39762, United States

[b] School of Environmental Sciences, Jawaharlal Nehru University, New Delhi 110067, India

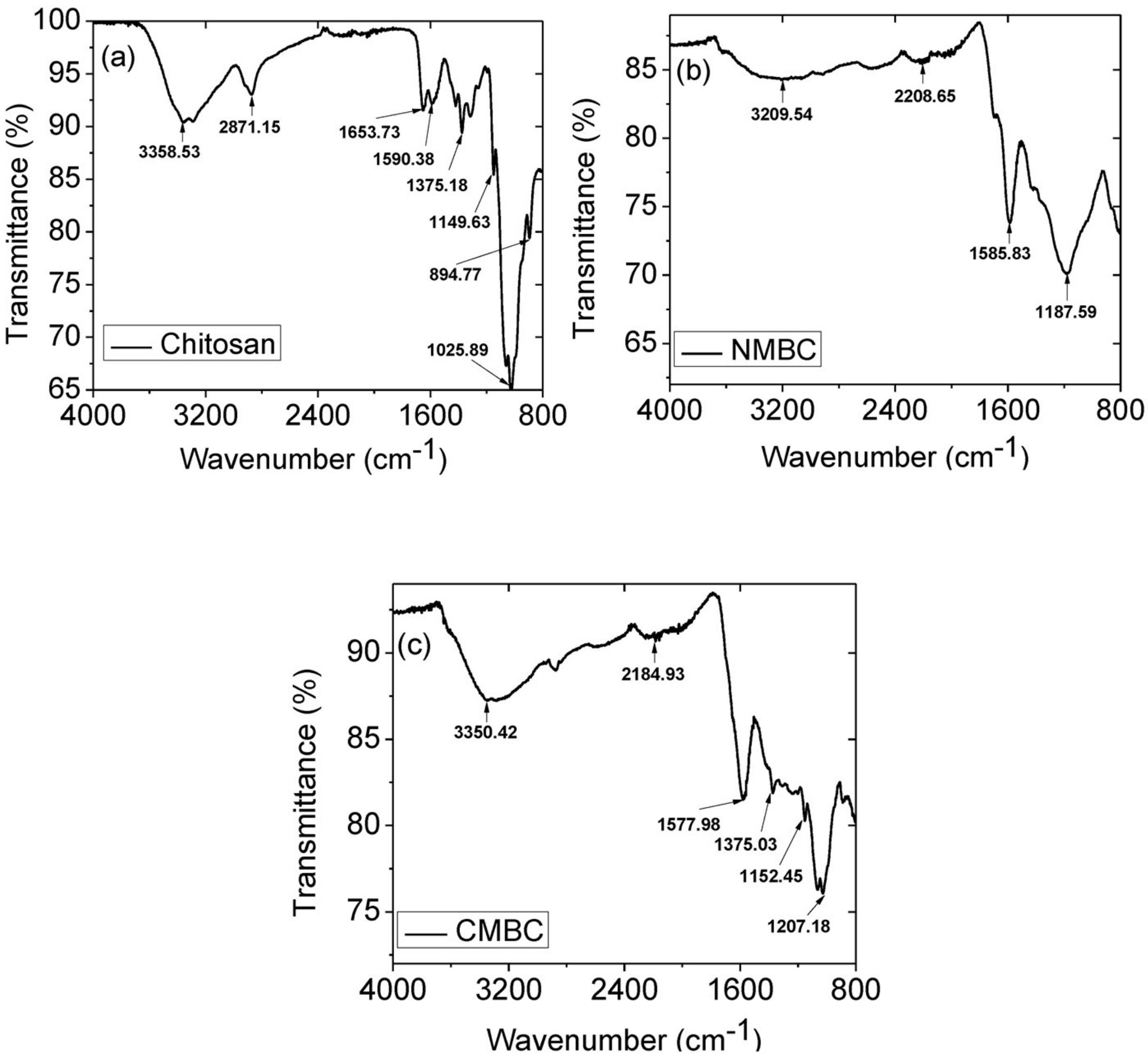

Fig. S1 FTIR spectra of (a) chitosan, (b) NMBC, and (c) CMBC.

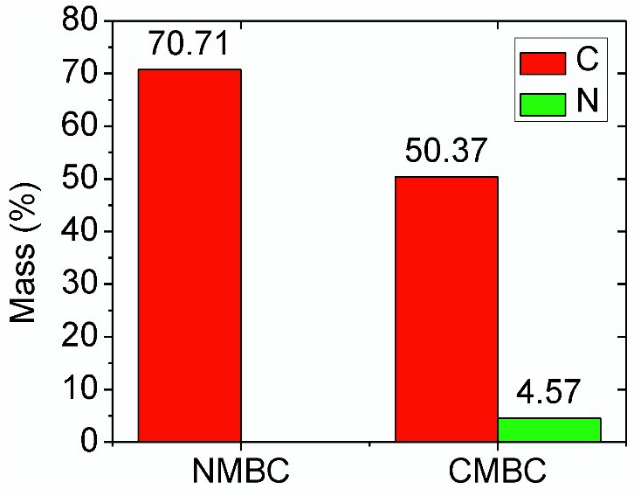

Fig. S2 C and N elemental analysis by combustion of NMBC and CMBC.

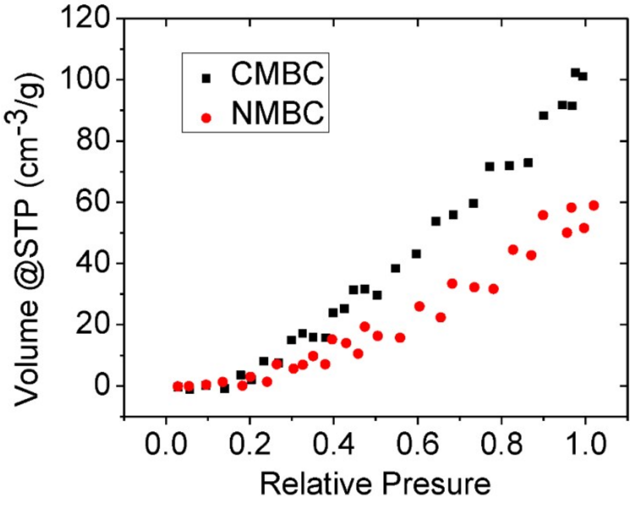

Fig. S3 N_2 adsorption isotherm of CMBC and NMBC.

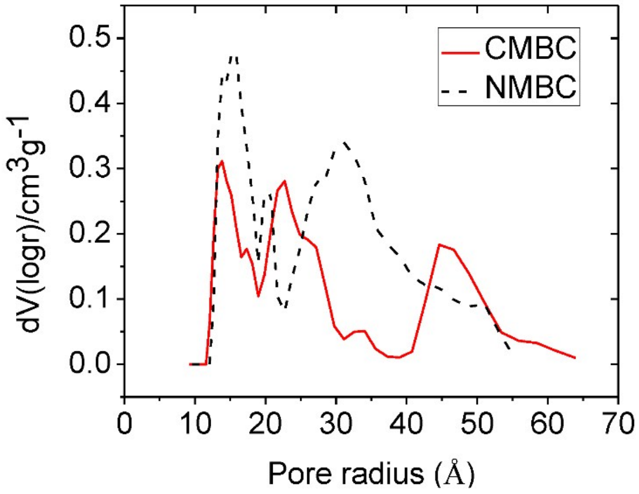

Fig. S4 Non-Local Density Functional Theory (NLDFT) pore size distribution curves of CMBC and NMBC.

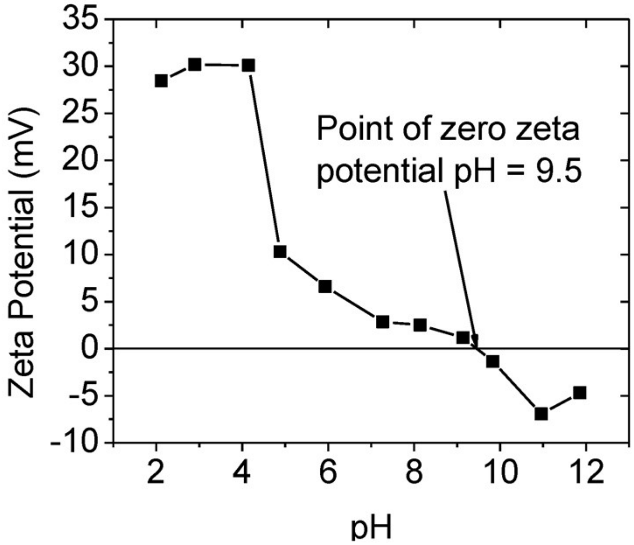

Fig. S5 The ζ-Potential of CMBC under different solution pH values.

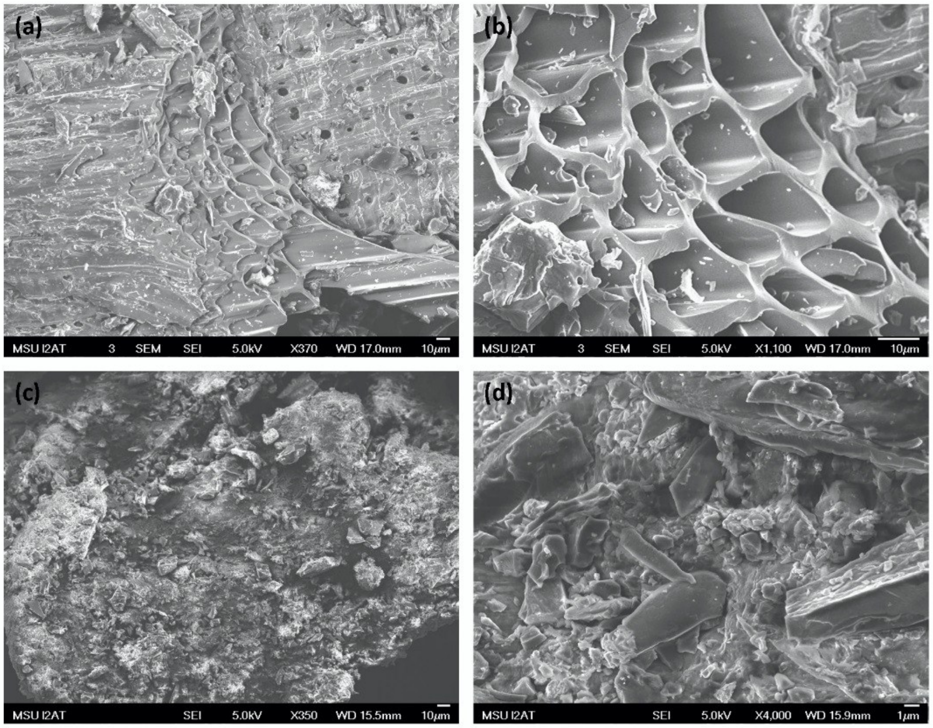

Fig. S6 SEM images of NMBC (a, b) and CMBC (c, d). The chitosan coating process appears to block some of the mesoporous cell surfaces including some pore openings.

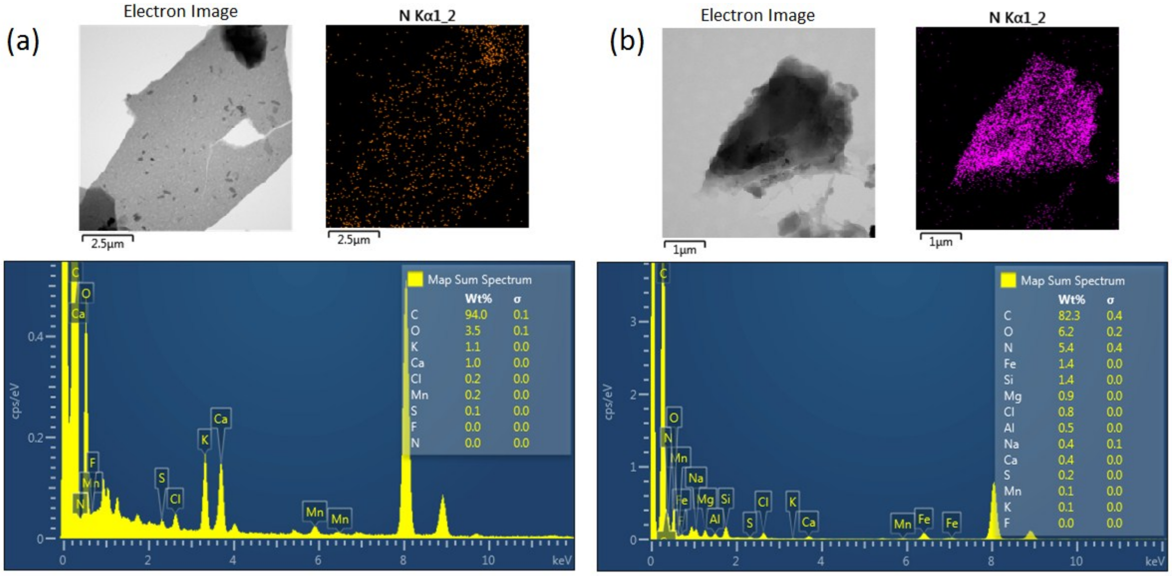

Fig. S7 Electron images, N elemental mapping, and EDX spectra of NMBC (a) and CMBC (b).

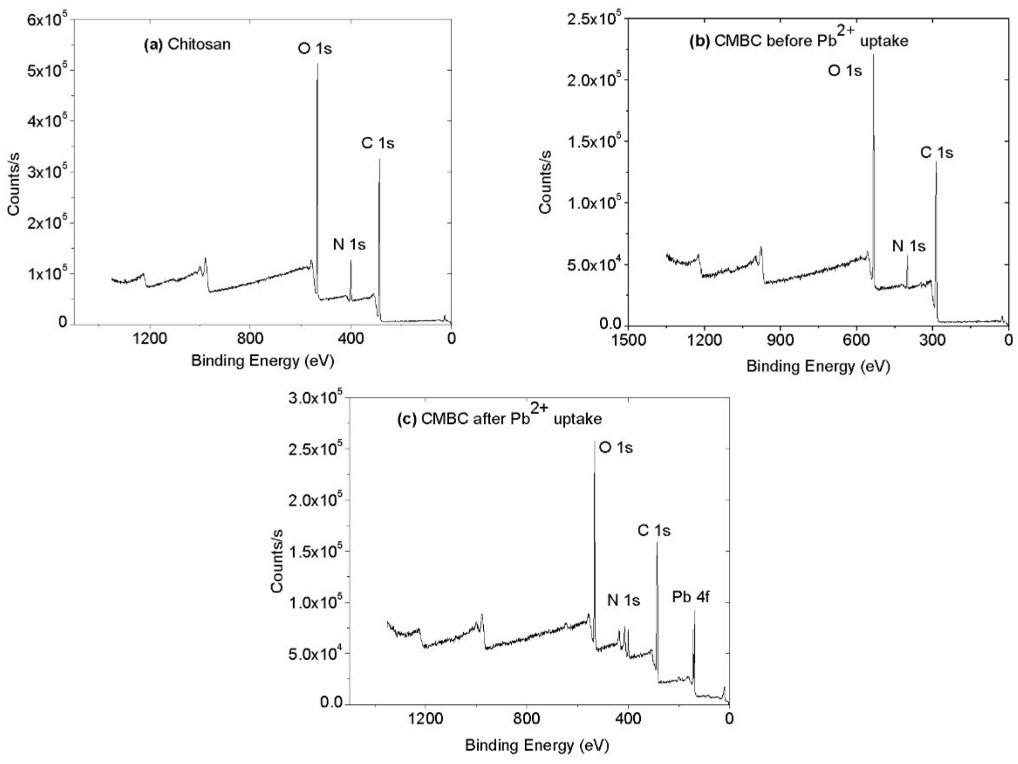

Fig. S8 Survey XPS spectra of (a) chitosan (b) CMBC before and (c) CMBC after lead adsorption.

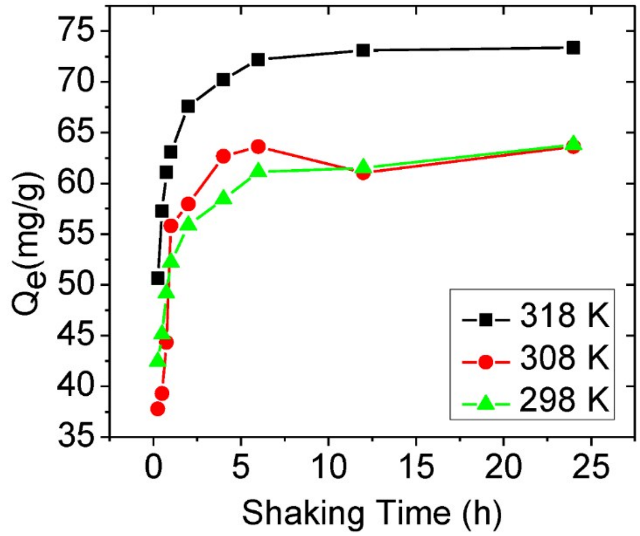

Fig. S9 Temperature effect on lead adorption at pH 5.0 and at 150 mg/L of lead solution using 2g CMBC/L.

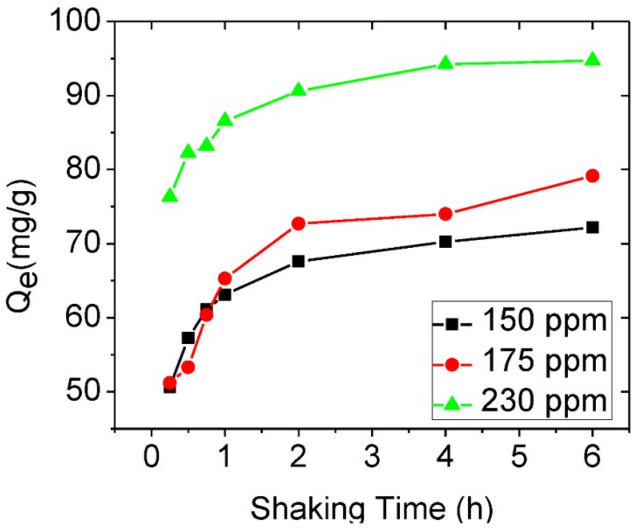

Fig. S10 The effect of the initial Pb concentration on capacity versus the shaking time at pH 5 using 2 g CMBC/L.

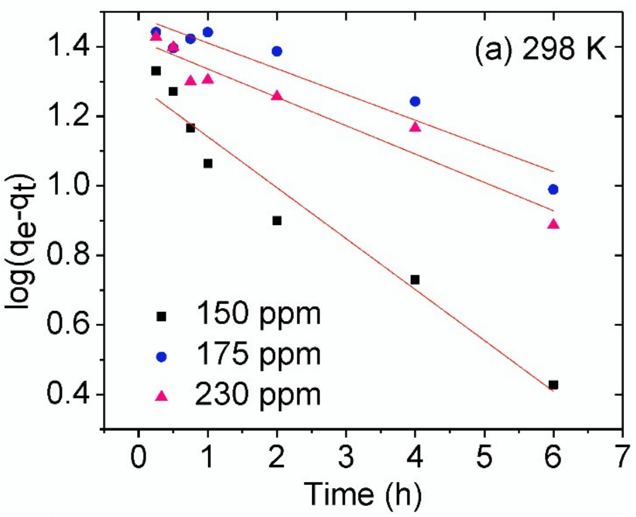
(a) 298 K
log(qe-qt)
Time (h)
150 ppm
175 ppm
230 ppm
1.4
1.2
1.0
0.8
0.6
0.4
0 1 2 3 4 5 6 7

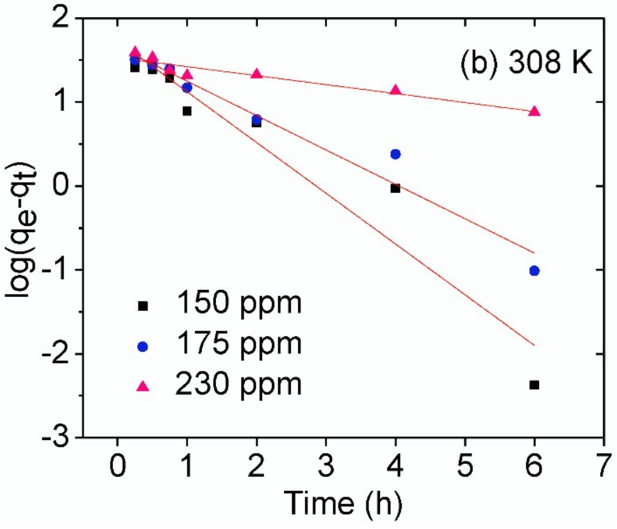
(b) 308 K
log(qe-qt)
Time (h)
150 ppm
175 ppm
230 ppm
2
1
0
-1
-2
-3
0 1 2 3 4 5 6 7

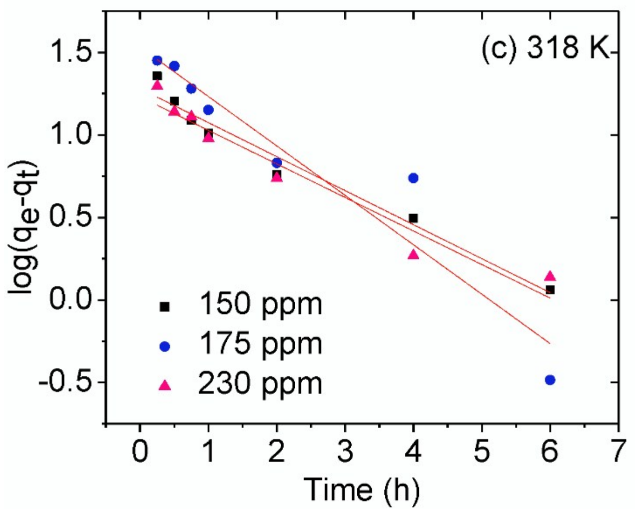
(c) 318 K
log(qe-qt)
Time (h)
150 ppm
175 ppm
230 ppm
1.5
1.0
0.5
0.0
-0.5
0 1 2 3 4 5 6 7

Fig. S11 Pseudo-first order plots for lead adsorption (pH=5) at (a) 298 K, (b) 308 K, and (c) 318 K for adsorbate concentrations of 150,175, and 230 mg/L.

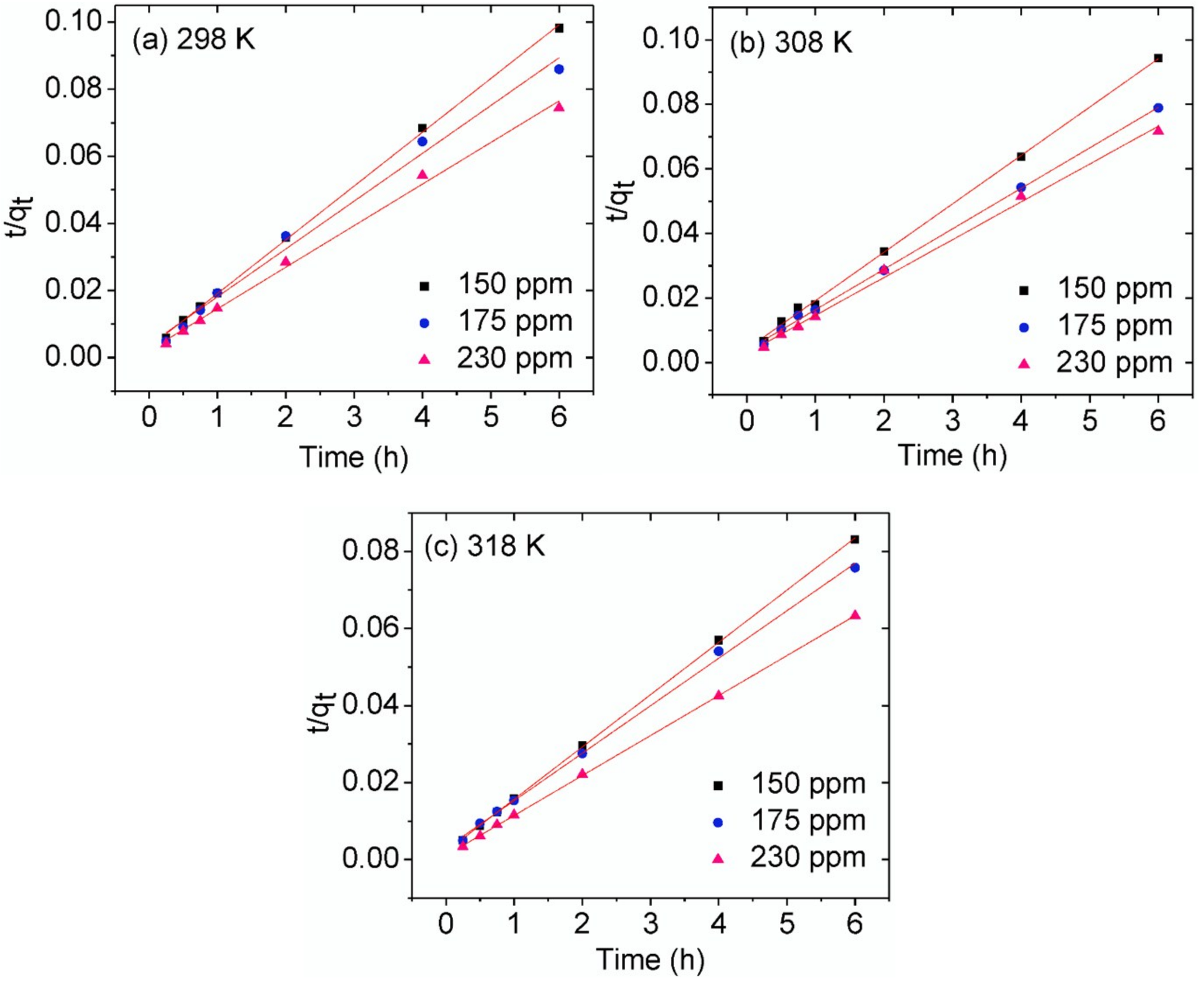

Fig. S12 Pseudo-second order plots for lead adsorption (pH = 5) at (a) 298 K, (b) 308 K, and (c) 318 K for Pb^{2+} concentrations of 150, 175, and 230 mg/L and 2 g CMBC/L.

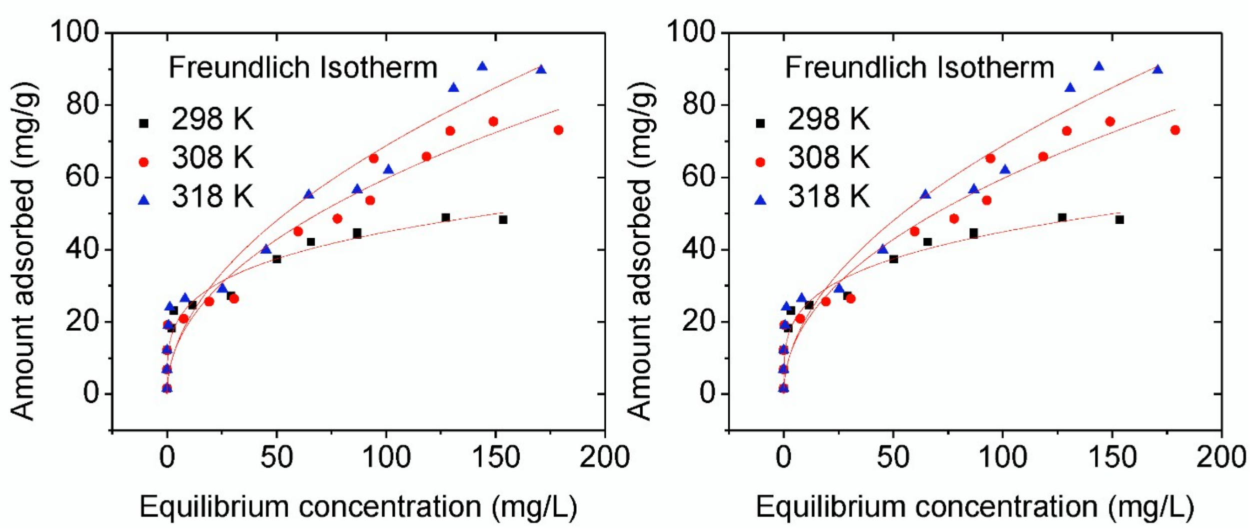

Fig. S13 Adsorption isotherms at 298, 308, and 318 K [pH = 5; adsorbent concentration = 2 g/L; Shaking time = 12 h].

Table S1 Combustion analysis, ash content, surface areas, and pore radius analysis for CMBC and NMBC

Sample	C (wt.%)	N (wt.%)	Ash content (wt.%)	Surface area (m^2/g)	Mesopore volume (cm^3/g)	Micropore volume (cm^3/g)	Total average pore volume (cm^3/g)	Density functional theory pore radius (nm)
NMBC	70.71	0.00	1.43	10.5	0.0911	0.000	0.091	1.385
CMBC	50.37	4.57	2.55	7.13	0.160	0.000	0.160	1.385

Table S2 The extent of protonation of chitosan at different pH values

pH	% extent of protonation
4.3	99
5.3	91
6.3	50
7.3	9

Table S3 Pseudo-first order parameters for lead adsorption (pH=5) at (a) 298 K, (b) 308 K, and (c) 318 K for adsorbate concentrations of 150, 175, and 230 mg/L

Pseudo-first order parameters					
Temp. (K)	Initial conc. (mg/L)	q_e exp. (mg/g)	q_e calc. (mg/g)	k_1 (g mg $^{-1}$ h^{-1})	R^2
	150	63.81	19.36	0.336	0.958
298	175	79.59	30.48	0.170	0.925
	230	88.32	26.12	0.187	0.934
	150	63.61	53.33	1.391	0.927
308	175	76.14	45.50	0.942	0.961
	230	91.34	33.81	0.244	0.918
	150	73.35	19.10	0.474	0.970
318	175	79.47	34.04	0.689	0.915
	230	96.09	17.02	0.468	0.948
